# Household Surgery, or Hints on Emergencies

**Published:** 1847-07-01

**Authors:** 


### Household Surgery, or Hints on Emergencies. By *John F. South,* 12mo. pp. 340. Cox, 1847.

The Translator of Chelius, late Professor of Surgery to the College of Surgeons, a Member of its Council, and one of the Surgeons of St. Thomas's Hospital, here makes his appearance in a new character, that of

Gossiper on Surgery with the non-medical public, and in that capacity has sent forth a very objectionable book. Popular works upon medical subjects are produced under two different influences: the one a desire on the part of the author to write himself into notice as a remarkably successful practitioner in the class of diseases he has chosen for illustration; the other in a foolish belief that the public at large possess sufficient intelligence and discrimination to successfully apply knowledge which it has taken a long special training to acquire—that there is, in fact, a royal road to the diagnosis and treatment of disease which has only to be pointed out to be pursued. However different may be the moral position of these two classes of authors, the mischievous effects of their proceedings are much the same; and it may be safely said that the world has suffered as much from the blunderings of fools as from the machinations of knaves. We cheerfully acquit Mr. South of any of those puffing designs which have presided over the production of so many Chest-books, Skin-books, Pox-books, and the like, and only charge him with a mere act of simpletonism—a charge which, small as is the space we have at our disposal, we doubt not being able to make good.

It seems the work originated in some popular lectures which its author delivered some time since to a rural audience. He says,

> "Though the subject might seem an odd one to select for a village auditory yet there was no difficulty in rendering it amusing as well as instructive, and I believe that I may justly say that I did not fail of attaining my object. On accidentally mentioning the circumstance to one of my friends, who, by means of the printing press, furnishes food to a hungry and never-satisfied public, he said, 'Why do you not make a little book of it? Do so; and I will print it.' So we struck hands, and here appears my small volume."

We are not much accustomed to the admeasurement of the risible faculties of village boors and Lady Bountifuls; but if they found anything amusing in the lectures, it must have been derived from the manner of the lecturer (but no: certain dreamy recollections of the theatre of the College of Surgeons forbid this conjecture), or it must have been of so subtle a nature as to ooze out during the process of printing; for nothing of the kind do we discover. As to the instruction aimed at, a pretty wide field has been embraced, if not compassed. The author commences his work with enumerating the various contents of what he terms "The Doctor's Shop" with which a family in the country should be provided, and among the "few simple family medicines with which no house should ever be unprovided," we find the following, calomel, tartar-emetic, iodide of potassium, blue-pill, opium, and croton oil; and among the formulæ given are those for making Goulard and Sugar of Lead Wash, Black-wash, Washes of Nitric Acid, Chlorine, and Oxide of Zinc; Croton-oil Embrocation; Red-precipitate, Iodide of Potassium, Lead, Gall, Zinc, and Sulphur Ointments; and these directed to be used "for the eyes," "sluggish sores," "sluggish sores with stinking discharge," "stinking ulcers or sloughy wounds," "cracked skin of the legs," "scabbing of large ulcers," "stimulating wounds or ulcers," "to lap up the discharge," "for enlarged glands of the neck," "for piles," "as a certain cure for the itch," as the case may be.

When treating of *Bleeding*, the author judiciously enough recommends, in the case of obstinate hæmorrhage from a leech-bite, that the orifices be taken up by means of a sewing-needle, but he adds that, in some constitutions, " even after the removal of the needle and thread the bleeding will continue. Nothing then remains but to thrust into the bottom of the wound a bit of thin iron wire heated white-hot, which I have never known fail to stop the bleeding." We would simply ask him whether he has ever met with a case requiring this, and if so, sufficiently often to justify his placing such a recommendation in such a book ? Cupping, we are told, has " the advantage of being easily learned," and, we may add, as easily forgotten unless constantly practised. To enable his amateur to acquire it, Mr. S. furnishes diagrams of the instruments and process. With regard to general bleeding, he does not consider the uninstructed should venture to bleed from the arm, but he may work away at the foot safely enough. The art and mystery of Tooth-drawing, whether by key or forceps, is dispatched in four pages (with appropriate diagrams), and then we come to a chapter upon " How to put on a Roller." We allow that the knowledge of this may be useful to many persons ; but we would suggest that one practical lesson from the surgeon who recommends its employment would do more than all Mr. South's pages and diagrams ; at all events we feel certain that we could never have learned to roll a leg from his description. The T bandage, " for keeping a poultice on the seat," is a simpler matter; and his lady-readers will understand his diagram well-enough. Of the mode of lancing the gums with a " gum-fleam" we have also an illustration : but, although we agree with Mr. South that the child will often in an hour or two afterwards derive immense benefit from the operation, we doubt whether the anticipation of this will induce it to hold its head erect and unsupported as here depicted. We do not know whether the following passage was intended for a moral hint to his rustic auditory, or as a specimen of dry humour.

" The greater number of persons who get a black eye deserve it, and so far as I am aware there is no remedy save warm-bathing which will hasten its removal; but it is often a very tedious business. The only thing to be borne in mind is not to get a black eye ; if you do, you must be content to bear the disgrace for a few days if you deserve it. But if it have been an accident, there is nothing to be ashamed of, and a small draught of patience will be a sovereign remedy."

Believing ourselves that a black-eye may be got quite undeservedly, and that its persistence is to be deprecated, we recommend our readers to discard our author's temporizing procedures, and continuously to apply slices of bryony root, or a coagulum formed of alum and white of egg between muslin, which will often rapidly dissipate it.

But we approach more serious matters. Thus we have detailed directions for treating *Wounds* of all kinds, whether a " clean stab," a " bruised cut," a " torn or rent wound," " pricks or punctured wounds," and wounds from serpents. For a bite from a mad-dog, the author is energetic enough, but not too much so, when there is no doubt of the animal's rabid condition. " Let a probe or piece of stick be passed down to the bottom of the wound, and insist upon the operator cutting around the wound, and so deeply as to bring out the probe or stick covered with the part cut out as with the finger of a glove." " I do not think I am wrong in advising, where the finger has been bitten, and there is no possibility of medical assistance, to chop it off, which any one can do with a mallet and chisel."

The variety of wounds might have been supposed to sufficiently tax the attention of the amateur surgeon; but no: he is here taught, and shown by woodcuts, the modes of treating rupture of the tendo-Achillis, nearly all the varieties of *Fracture* (compound and simple), and the mode of reducing *Dislocations*, from that of the jaw to that of the hip. In respect to *Hæmorrhage*, we have directions for treating hæmoptysis (free bleeding among them) and hæmatemesis, for taking up arteries in wounds, and the compression of the large trunks. For operating for *Rupture*, the presence of a surgeon is admitted to be necessary, but, in the mean time, the taxis, the application of cold, bleeding to faintness, and turning the patient topsy-turvy may be resorted to.

We have enumerated nothing like the whole of the contents of the book. The various forms of "Stifling" by carbonic acid, drowning, hanging, &c. are treated of diffusely, and there are chapters on Things in the Gullet, Windpipe, Nose and Ears. A chapter is added upon the dress, diet, and exercise of children, in which these subjects—the only ones almost the public can be advantageously addressed upon—are most superficially treated; and another treats of the prevention of infectious fevers by ventilation and the non-crowding of the sick, in which nothing is said which has not been better said a hundred times before.

In fine, we may state of this book, that most of the subjects it embraces are utterly unsuited for general description and illustration: and those which admit of these the author has shewn himself incompetent to treat of. It were unjust, however, to dismiss it without adverting to an excuse he offers for its publication. He explains the addition of the portions which relate to wounds and broken limbs, &c. by the suitableness of such information to persons at sea or in commencing colonies, unprovided with medical advice. We are not prepared to say that a pamphlet addressed to this comparatively small class of persons might not have its uses; but this book bears no designation of the kind, and was doubtlessly intended for circulation in this country, in no part of which is medical aid unattainable. The author has some misgivings of the reception which awaits him; for he says:

> "It is very possible that some observations may be made on a Hospital Surgeon writing a book of this kind, intended for general use. I am very careless on this point, as I have had no unworthy object in view. The way in which, and the purpose for which the book has been written, are my apology, if any be needed, which I do not admit; and if I desired precedent I need scarcely remind my readers how many of the ablest persons in science and art have held it no degradation to their high standing to render their particular branch of knowledge accessible, not only to adults but even to children, by cheerfully written works, in simple language, often accompanied with homely illustration."

We do not admit that good intentions form a sufficient excuse for injurious actions. If they exculpate the individual from moral reprobation it is at the expense of his capacity. Nor will the precedents here alluded to hold good. Many of the objects of science and art admit of familiar and popular illustration; and the worst result which can ensue from their partial comprehension is the generation of smatterers and ridiculous pseudo-savants; but it is a graver matter with regard to medicine. In the first place the facts are more difficult of summary communication and comprehension; and the ill-consequences of their imperfect acquisition are not limited to the individual dabbling with them, but may injuriously affect many other portions of society. Hence it is the duty of all members of our profession to discountenance instead of encouraging such pretensions; and we are of opinion that the egregious manner in which Mr. South has violated such a duty should, *ipso facto,* forfeit the responsible seat he holds at the board of one of its governing bodies.

